# Supplementary material for: Modality specific roles for metabotropic GABAergic signaling and calcium induced calcium release mechanisms in regulating cold nociception
Source: Front Mol Neurosci. 2022 Sep 9;15:942548. doi: 10.3389/fnmol.2022.942548 (PMC9502035; doi:10.3389/fnmol.2022.942548)
Supplement: Supplementary file 1 [file Data_Sheet_1.PDF]

Supplementary Material

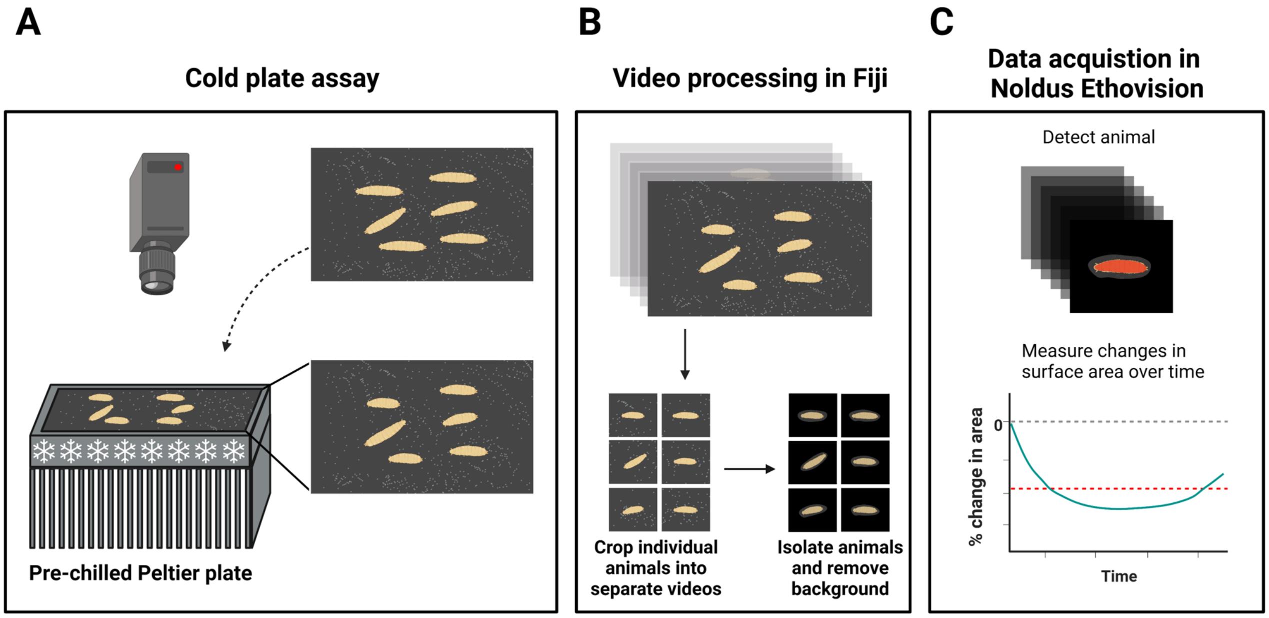

Supplementary Figure 1: Cold plate assay schematic. (**A**) Graphical representation of cold plate assay. Freely moving third instar larvae on black metal plate are exposed to noxious cold stimulus by placing the plate on pre-chilled Peltier surface. (**B**) Video processing pipeline in FIJI. Original video is trimmed from stimulus delivery to 5 seconds. Trimmed video is cropped into multiple videos to contain one larva per video. Background is removed from cropped videos for greater signal to noise ratio. (**C**) Acquisition of changes in larval surface area over time measurements are performed using Noldus Ethovision XT. Schematic created with BioRender.com.

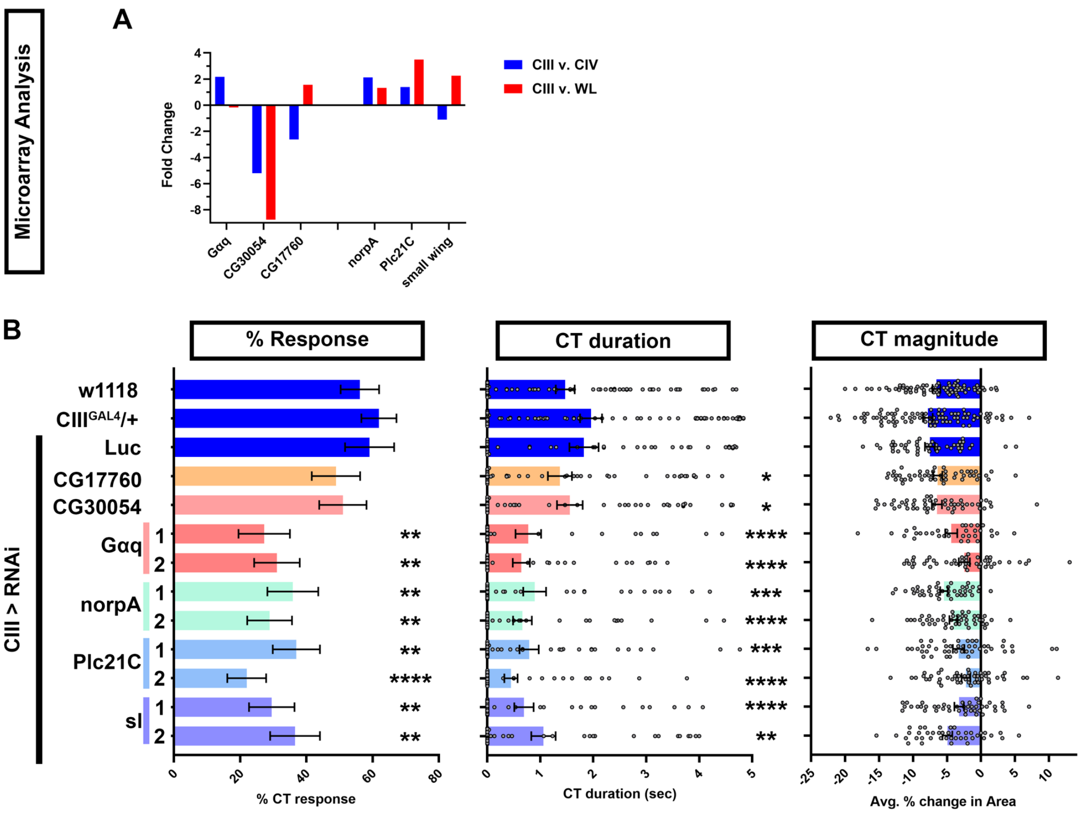

Supplementary Figure 2: Gαq and phospholipase C required for cold evoked CT response in CIII md neurons.

(**A**) Fold change in expression for $G_{\alpha q}$ ($G_{\alpha q}$, *CG30054, CG1760*) and *PLCs* (*norpA*, *Plc21C*, and *small wing* (*sl*)) comparing CIII md neuron and CIV md neurons or whole larva (WL) microarray datasets. (**B**) Cold plate analysis of *Drosophila* larvae with CIII md neuron specific gene knockdown of $G_{\alpha q}$ and *PLCs*. We report %CT (left), CT duration in seconds (middle), CT magnitude (right). Control conditions include: *w1118*, *19-12*^{GAL4}/+, and *19-12>Luc*^{RNAi}. There is no statistical difference between the controls. Knockdown of $G_{\alpha q}$ and *PLCs* comparisons were made to *19-12*^{GAL4}/+. $N_{Average}$ = 49. Significant differences indicated via asterisks, where *p<0.05, **p<0.01, ***p<0.001, ****p<0.0001.

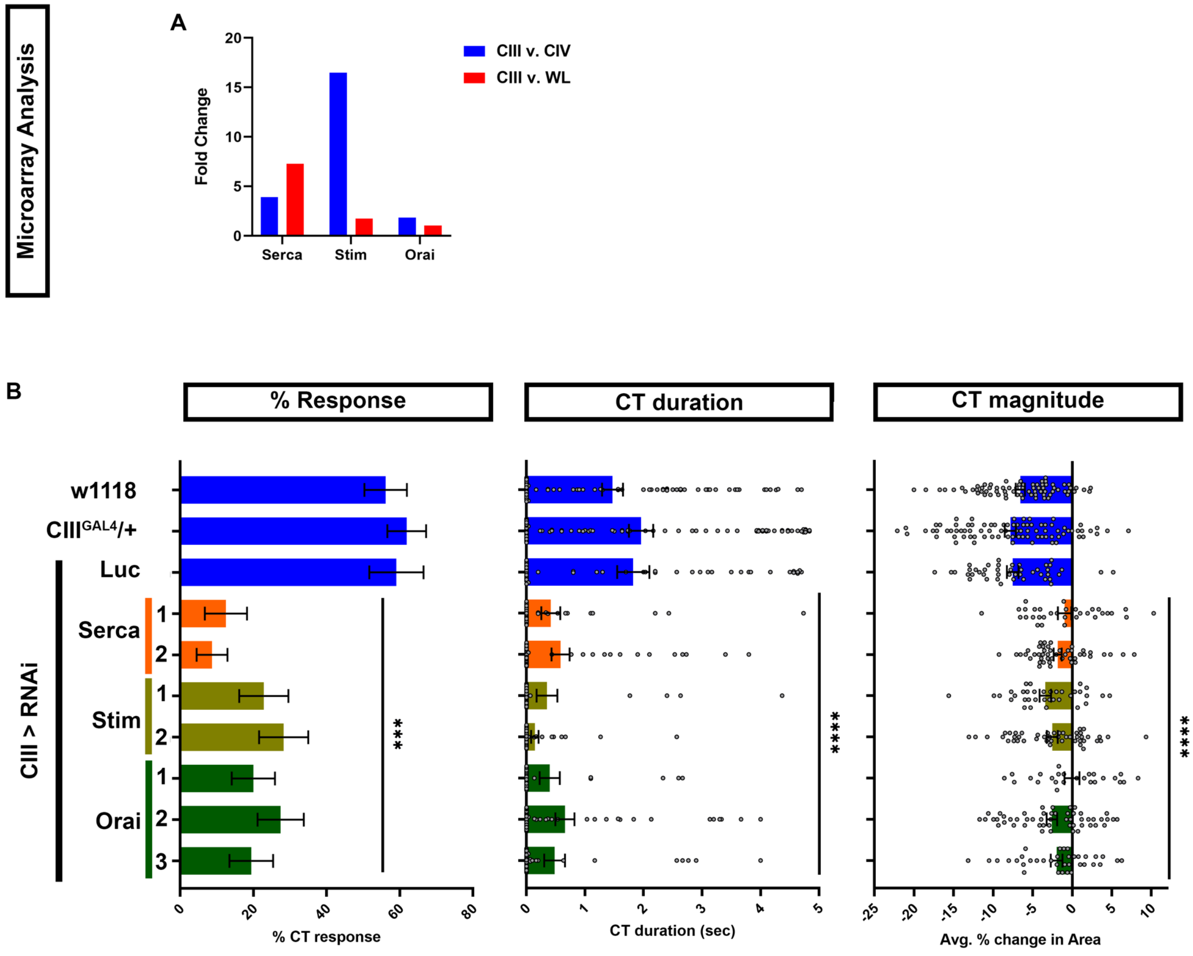

Supplementary Figure 3: Requirement for Ca^{2+} reuptake genes in cold evoked CT behavior.

(**A**) Microarray analysis of *SERCA*, *Stim* and *Orai* in CIII md neurons compared to CIV md neurons or whole larva (WL). (**B**) Ca^{2+} reuptake gene knockdown specifically in CIII md neurons using *19-12^{GAL4}* driver. We report %CT (left), CT duration in seconds (middle), CT magnitude (right). Control conditions include: *w1118*, *19-12^{GAL4}/+,* and *19-12>Luc^{RNAi}*. There is no statistical difference between the controls. Knockdown of *SERCA*, *Stim* and *Orai* was tested with at least two independent RNAi lines and comparisons made to *19-12^{GAL4}/+*. $N_{Average} = 47$. Significant differences indicated via asterisks, where *p<0.05, **p<0.01, ***p<0.001, ****p<0.0001.

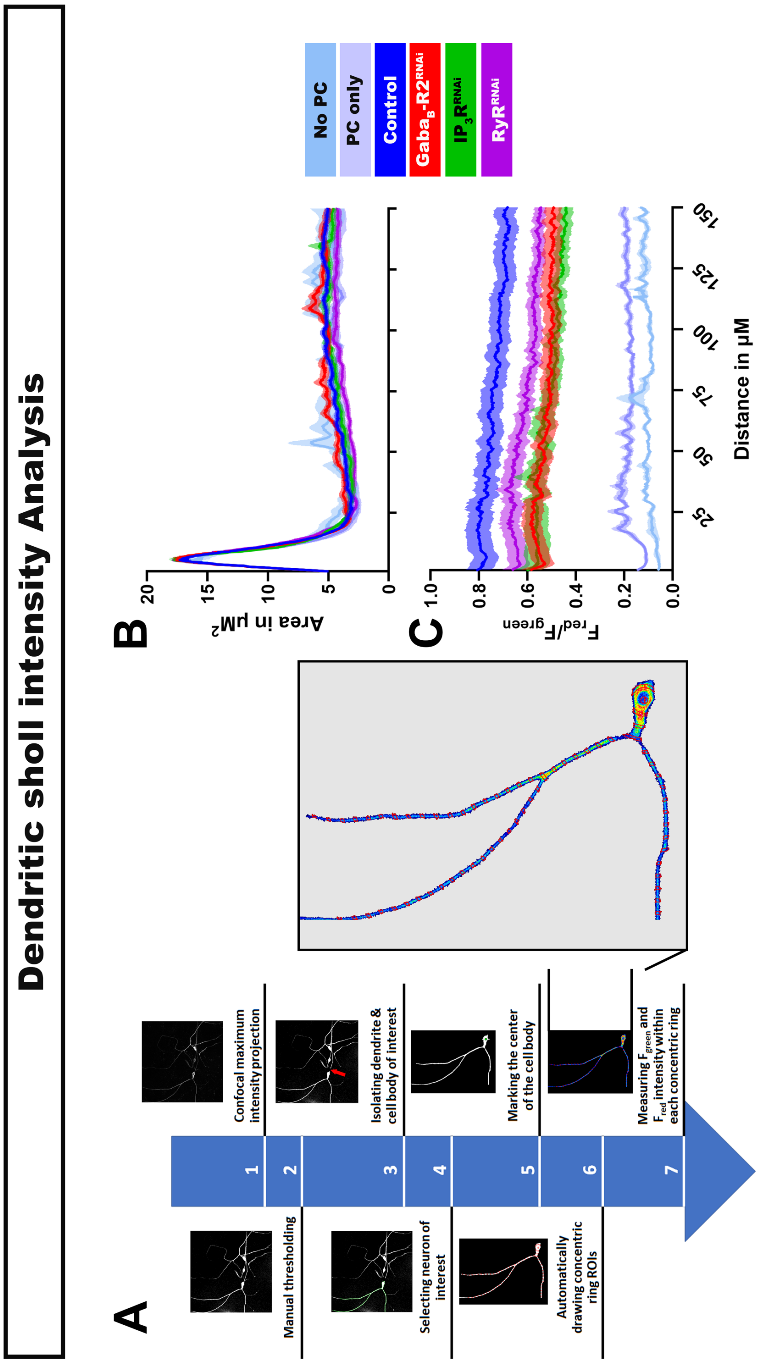

Supplementary Figure 4: 2D Sholl intensity analysis of dendritic CIII md neuronal CaMPARI2 response.

(**A**) Schematic of custom FIJI macro script for rapidly removing background, selecting cell body and dendrites of interest, automatic generation of sholl ROIs, and measuring area normalized F_{red} and F_{green} intensities. (**B**) Average area in μm^2 for individual ROIs as function of distance from the center of the cell body. (**C**) Area normalized average F_{red}/F_{green} ratio of CaMPARI2 plotted as a function of distance away from the cell body. $N_{Average} = 30$.

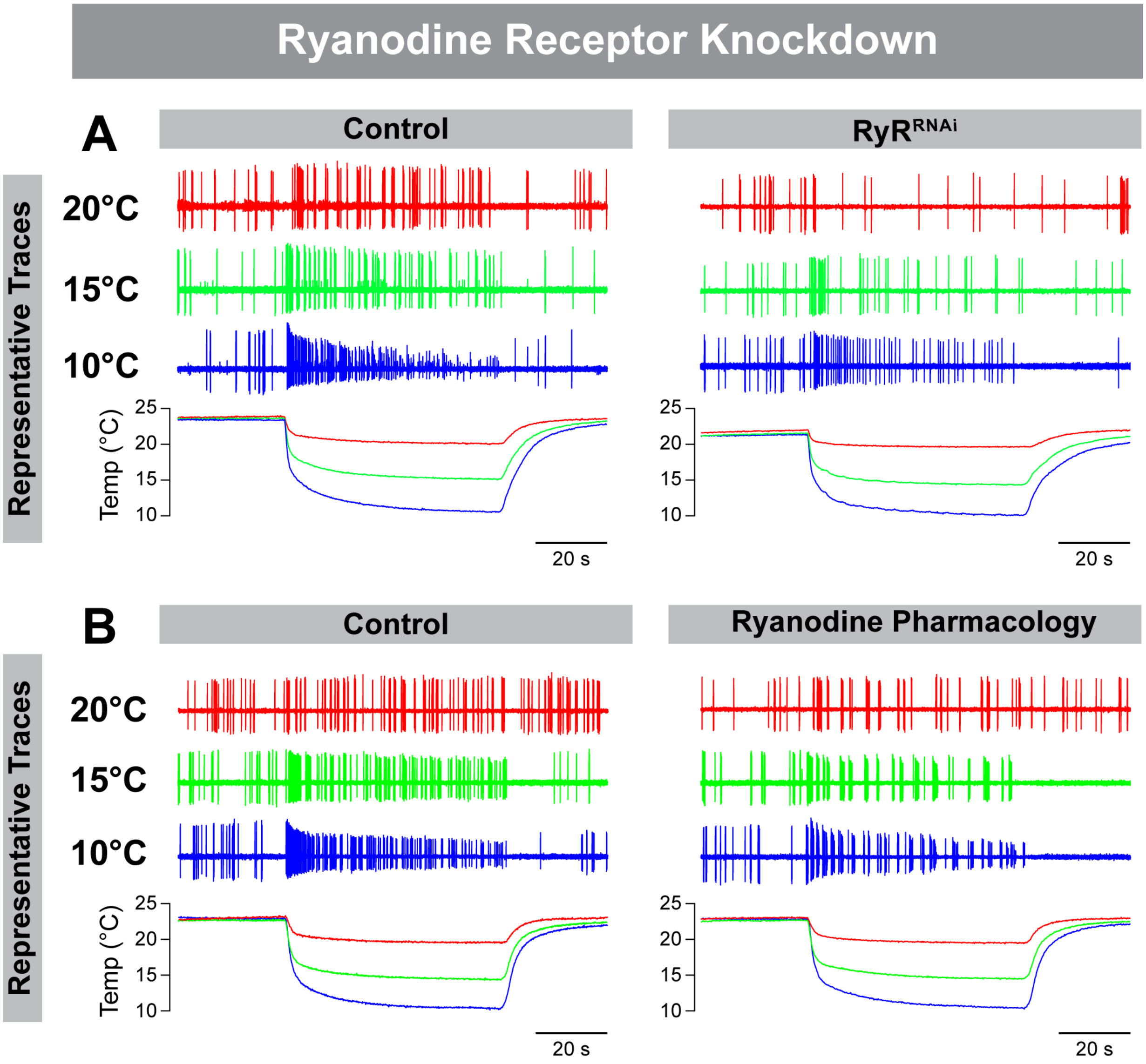

Supplementary Figure 5: Representative extracellular recording traces CIII md neurons.

Representative physiological firing traces for each stimulus temperature (20°C, 15°C, 10°C) and below shows real time temperature exposure regimes of *Drosophila* larval filet. (**A**) *RyR* knockdown compared to controls. (**B**) Pharmacological application of ryanodine (10^{-6} M) compared to controls.

Supplementary Table 1: List of Drosophila melanogaster strains

Designation	Source or reference	Identifier
w1118	BDSC	3605
***19-12**^{GAL4}*	Yan et al. (2012). *Nature* 493:221-225	
***GMR83B04**^{GAL4}*	BDSC	41309
***nompC**^{GAL4}*	BDSC	36361
UAS-CD4-tdGFP	BDSC	35836
UAS-CaMPARI2	BDSC	78317
UAS-IVS-GCaMP6m	BDSC	42748
UAS-ChETA::YFP	BDSC	36495
Luc RNAi	BDSC	31603
GABA-B-R1 RNAi - 1	BDSC	28353
GABA-B-R1 RNAi - 2	BDSC	51817
GABA-B-R2 RNAi - 1	BDSC	50608
GABA-B-R2 RNAi - 2	BDSC	27699
GABA-B-R3 RNAi - 1	BDSC	26729
GABA-B-R3 RNAi - 2	BDSC	50622
GABA-B-R1 MiMIC	BDSC	44860
GABA-B-R1 KO - CRISPR	BDSC	84503
GABA-B-R1 KI - CRISPR	BDSC	84701
GABA-B-R2 MiMIC	BDSC	59503
GABA-B-R2 KO - CRISPR	BDSC	84504
GABA-B-R2 KI - CRISPR	BDSC	84634
GABA-B-R3 MiMIC	BDSC	23554
GABA-B-R3 KO - CRISPR	BDSC	84505
GABA-B-R3 KI - CRISPR	BDSC	84635
Gαq RNAi - 1	BDSC	36820
Gαq RNAi - 2	BDSC	36775
CG17760 RNAi	BDSC	64919
CG30054 RNAi	BDSC	51283
norpA RNAi - 1	BDSC	31113
norpA RNAi - 2	BDSC	31197
Plc21C RNAi - 1	BDSC	31269
Plc21C RNAi - 2	BDSC	33719
sl RNAi - 1	BDSC	32385
sl RNAi - 2	BDSC	32906
IP3R RNAi - 1	BDSC	51795
IP3R RNAi - 2	BDSC	51686

IP3R RNAi - 3	BDSC	25927
RyR RNAi - 1	BDSC	31695
RyR RNAi - 2	BDSC	31540
RyR RNAi - 3	BDSC	28919
RyR RNAi - 4	BDSC	29445
IP3R 90B0	BDSC	30737
IP3R ug3	BDSC	30738
IP3r ka901	BDSC	30741
RyR 16	BDSC	6812
RyR Q3878X	BDSC	5501
RyR Y4452X	BDSC	5507
Serca RNAi - 1	BDSC	25928
Serca RNAi - 2	BDSC	44581
Orai RNAi - 1	BDSC	53333
Orai RNAi - 2	VDRC	GD12221
Stim RNAi - 1	BDSC	27263
Stim RNAi - 2	BDSC	51685
Stim RNAi - 3	BDSC	52911
PKD2[1] mutant	BDSC	24495
